# Electrocortical and Physiological Correlates of Memory and Attention During the Anticipation of Avoidable and Inevitable Threats

**DOI:** 10.1111/psyp.70413

**Published:** 2026-09-27

**Authors:** Yannik Stegmann, Matthias Gamer

**Affiliations:** ^1^ Department of Psychology University of Würzburg Würzburg Germany

**Keywords:** alpha, attention, attentive immobility, avoidance, EEG, memory, threat

## Abstract

When confronted with danger, humans display a range of defensive behaviors, such as freezing and active avoidance. Prior research has identified a distinct physiological response pattern to avoidable threats, characterized by heart rate bradycardia, reduced visual exploration, and suppressed visual alpha activity (8–13 Hz), all of which are associated with faster flight responses. While this pattern indicates an adaptive state of attentive immobility, it is largely unclear how this state influences the encoding of explicit threat‐related memories. Clarifying these mechanisms seems important to understand the processes contributing to the formation of pathological fear memories. To address this question, we recorded parieto‐occipital alpha activity, eye movements, and autonomic responses in 60 participants during anticipation of either an avoidable threat, an inevitable threat, or no threat, while viewing images of various facial identities. In a subsequent memory task, participants were asked to recognize previously seen faces among novel ones. Results showed enhanced suppression of alpha activity during avoidable threats, accompanied by heart rate bradycardia, centralized gaze, and increased sympathetic arousal. However, these responses were not linked to memory encoding. Instead, memory effects were associated with reduced pupillary responses, heightened visual exploration, and decreased alpha activity, particularly in occipitotemporal regions. Together, these findings suggest that when individuals face avoidable threats, they enter a state of attentive immobility that enhances perceptual processing and prepares them for action but does not extend to improved memory encoding.

## Abstract 2

This study reveals that avoidable threats induce a distinct defensive state of attentive immobility, characterized by alpha suppression, bradycardia, and focused gaze that enhances perceptual readiness. Surprisingly, this state does not improve memory encoding. Instead, memory performance depends on separate autonomic and electrocortical responses. These findings clarify how humans adaptively balance rapid action preparation with longer‐term information processing, offering new insights into the neurocognitive organization of defensive behavior.

## 1 Introduction

Defensive behaviors are fundamental for effectively navigating dangerous environments. The ability to recognize and predict potential dangers is crucial for survival, as it allows organisms to avoid harmful situations (Fanselow 2018). At the same time, a dysfunction in these processes is discussed as a key factor for the development of anxiety disorders and can lead to excessive threat‐related responses, including hyperarousal and persistent avoidance behavior (Mineka and Oehlberg 2008). Memory processes play a central role in this context, as the encoding, consolidation, and retrieval of threat‐related experiences shape how future situations are interpreted and responded to.

Defensive behaviors engage an organism's full range of physiological and cognitive systems and can be organized along a threat imminence continuum (Blanchard et al. 1993; Fanselow 2018; Lang et al. 2000). During the pre‐encounter stage, the organism enters an environment where threats have been encountered previously, although no immediate danger is present. This stage is characterized by risk assessment, cardiac defensive mobilization, and heightened vigilance. Once a threat is detected, the organism transitions to the post‐encounter stage, marked by preparation for potential fight‐or‐flight responses. This stage involves freezing behavior, fear‐related bradycardia, and selective attention toward the threat (Lang et al. 2000). As direct contact to the threat becomes inevitable, the organism enters the circa‐strike stage, during which it engages in active fight or flight behaviors, accompanied by elevated sympathetic arousal and accelerated heart rate (Hamm 2020). Thus, the three stages of the threat imminence continuum reflect a tightly integrated interplay of autonomic nervous system activity, shifts in attentional processing, and transitions from freezing to fight‐or‐flight behaviors. A characteristic pattern of coordinated changes across these three domains has recently been described as the state of attentive immobility. Upon threat detection, the organism engages in freezing behavior accompanied by cardiac deceleration, which serves to reduce detectability by predators. Simultaneously, perceptual processing and motor readiness are enhanced, highlighting that attentive immobility is not merely a passive, but an active, adaptive state aimed at optimizing defensive responses in the face of impending danger (Roelofs 2017; Roelofs and Dayan 2022).

While original evidence for attentive immobility in response to threat detection was largely derived from animal work, recent studies have begun to explore its underlying mechanisms in humans. Given that attentive immobility appears especially relevant in contexts where threats can be actively avoided, these studies have focused on comparing defensive mechanisms between avoidable and inevitable threat conditions (Hashemi et al. 2019; Löw et al. 2015; Rösler and Gamer 2019; Wendt et al. 2017). This approach extends beyond traditional fear conditioning paradigms, where participants are typically instructed to passively observe stimuli without the possibility of active engagement. Using an approaching threat paradigm, Löw et al. (2015) found that skin conductance responses, an index of sympathetic arousal, increased with increasing imminence of an avoidable threat. In contrast, heart rate showed an initial acceleration followed by a deceleration just before the avoidance response became possible. This pattern mirrored startle response modulations, which exhibited initial potentiation followed by inhibition immediately prior to the opportunity for avoidance. A similar response pattern emerged in our previous studies (Merscher and Gamer 2023; Merscher et al. 2022; Rösler and Gamer 2019), which employed a design where a color cue at the beginning of each trial signaled whether an upcoming naturalistic image would be followed by an inevitable, avoidable, or no shock. Specifically, the phase preceding the avoidance response was marked by increased skin conductance and pupillary dilation, along with heart rate deceleration. Beyond autonomic nervous system measures, participants also showed reduced visual exploration during threat anticipation, suggesting freezing‐like behavior (“freezing of gaze”). This convergence of somato‐visceral and behavioral responses supports the concept of attentive immobility. Moreover, the magnitude of these responses predicted trial‐by‐trial variation in avoidance response times.

To further investigate the role of attention during attentive immobility, we recently adapted this paradigm to assess changes in alpha activity as an electrocortical marker of attentional engagement (Stegmann et al. 2024). In a sample of *n* = 101 healthy participants from Germany and the United States, we observed enhanced suppression of alpha activity not only in response to cue onsets but also toward the end of the trial (i.e., directly preceding the response) in the avoidable compared to inevitable threat condition. Moreover, the magnitude of alpha suppression predicted response times in avoidable threat trials, further highlighting the role of attentional processes in action preparation during states of attentive immobility. These findings are complemented by results from Martín et al. (2025), who demonstrated that heart rate deceleration in response to avoidable aversive stimuli was associated with increased steady‐state visual evoked field (ssVEF) amplitude, indicative of enhanced visual sensory processing, and reduced beta‐band desynchronization over frontal electrode sites contralateral to the response hand. Given that beta‐band desynchronization reflects preparatory activation of motor‐related cortical circuits, its attenuation suggests reduced motor readiness. Together, these results support the notion that attentive immobility involves a coordinated state of heightened perceptual engagement and suppressed motor preparation, optimizing sensory intake in the context of threat anticipation.

Recently, studies have begun to explore the impact of attentive immobility on higher‐order cognitive functions, such as decision‐making under threat (Klaassen et al. 2024). Yet, despite its potential to shape how threat‐related information is encoded and later retrieved, the influence of attentive immobility on declarative memory processes remains largely unexplored. Since much of the freezing literature is based on animal models, these declarative memory processes are often overlooked, even though they play a crucial role in treating pathological anxiety, where cognitive‐behavioral therapy frequently targets explicit, declarative memories (Hofmann 2008). It is well established that emotional stimuli are remembered better than neutral stimuli (Bradley et al. 1992; Dolcos and Cabeza 2002; Ventura‐Bort et al. 2016). However, the underlying mechanisms of threat‐related declarative memory modulation remain elusive. Although physiological arousal appears to play a crucial role in explicit memory formation, interpretations are not straightforward, as previous studies have yielded mixed results, ranging from enhanced retrieval (Ventura‐Bort et al. 2016) via absent memory modulation (Schellhaas et al. 2020, 2022) to decreased memory performance (for a general review, see Mather and Sutherland 2011). To explore the neurophysiological correlates of memory formation, studies typically employ a Subsequent Memory Task (Hanslmayr and Staudigl 2014; Paller and Wagner 2002) that contrasts neural activity during encoding between items that are later remembered and those that are not. The differences in activity that distinguish between successfully remembered and forgotten items (subsequent memory effects) reflect the specific neurophysiological processes that facilitate later retrieval. Recent studies have combined aversive learning with subsequent memory paradigms while recording EEG to explore the electrocortical correlates of threat‐related memory encoding (Leimeister et al. 2023; Wiemer et al. 2024, 2021). These studies showed that the same electrocortical components associated with attentional mechanisms, such as the P300, LPP, and alpha‐band activity, also played a crucial role in encoding declarative threat and safety memories. In summary, these findings suggest that activation of the defense system not only engages attentional processes and prepares the organism for action but also serves the long‐term goal of forming a sustained memory trace of potential threats, thereby facilitating adaptation in future situations. However, direct links between a state of attentive immobility and declarative memory processes have not yet been investigated.

Consequently, the goal of the current study was to investigate the effect of attentive immobility on memory processes. To this end, facial stimuli were presented as task‐irrelevant environmental information during the anticipation of avoidable or inevitable threats, or during periods of safety, allowing us to assess incidental memory formation under different defensive states. In a subsequent memory test, participants were shown previously seen faces alongside new ones and were asked to indicate whether they had seen the face before. We expected that the increased suppression of alpha activity and enhanced physiological responses during states of attentive immobility in avoidable threat trials would also lead to better memory performance.

## 2 Method

### 2.1 Participants

All hypotheses, methods, and analyses were preregistered in October 2024 at https://osf.io/meztd. A total of 64 participants completed the study. However, four participants were excluded from all analyses due to a combination of memory results and artifacts in the EEG signal, resulting in empty cells in at least one condition (see below for details on participant exclusion criteria). This led to a final sample size of *N* = 60 (23.42 ± 3.58 years old, 77% female, 6% diverse). We determined a target sample size of *n* = 60 based on a power analysis informed by previous experiments. For the critical comparison of avoidable threat versus safety during the onset of visual stimuli, we obtained an effect size of Cohen's *d* = 0.32 in our sample (Stegmann et al. 2024). For memory effects, Leimeister et al. (2023) reported a Cohen's *d* = 0.36 for differences in alpha‐band activity between remembered and forgotten faces. Using the *pwr* package (version 1.3‐0) in R, we conducted a power analysis based on the smaller of these effect sizes (*d* = 0.32), corresponding to the effect of avoidable threat anticipation on alpha activity. This analysis indicated a required sample size of *n* = 61 to achieve 80% power at an alpha level of 0.05. With our planned sample size of 60, a comparable power analysis for the memory effect on alpha amplitudes (*d* = 0.36) yielded a power of 0.86. All participants were older than 18 years and had normal or corrected to normal vision (with contact lenses only). None of them indicated previous pain‐related diseases. Participants received compensation in the form of class credits or money (12 € per hour) for their voluntary participation. Before their participation, they provided written informed consent according to the recommendations of the Declaration of Helsinki. The study design and all methods were approved by the local ethics committee of the University of Würzburg. Data were collected between October 2024 and January 2025.

### 2.2 Stimuli and Apparatus

As visual stimuli, we used 171 black and white photographs of young, middle‐aged, and older males and females with neutral facial expressions, obtained from the FACES database (Ebner et al. 2010). For each identity, we also included the corresponding image with an angry expression (see Section 2.3 below). For each participant, 90 of the neutral images were randomly selected and assigned to one of the three experimental conditions. Each selected image was presented three times to the participant, always in the same condition. For the memory task, an additional 60 novel neutral images were randomly selected from the remaining dataset. The stimuli were presented in the center of the screen in front of a gray background, using a 27‐in. LCD monitor with a resolution of 2560 × 1440 pixels and a refresh rate of 60 Hz. At a fixed viewing distance of 80 cm, the images spanned visual angles of 8.7° horizontally and 11.0° vertically. For stimulus presentation, the experimental software *Presentation* (Neurobehavioral Systems Inc., Albany, CA, version 23.0) was used.

The unconditioned stimuli (US) consisted of a 150 ms aversive electrical pulse train (three 2 ms pulses, separated by 48 ms) and were delivered to the inner side of the left forearm through surface bar electrodes consisting of two stainless‐steel disks of 9 mm diameter and 30 mm spacing by a constant current stimulator (Digitimer DS7A, Digitmer Ltd., Welwyn Garden City, UK). Similar to our previous work (Stegmann et al. 2022), US intensities were individually adjusted, using a staircase‐procedure consisting of two ascending and descending series of electrical stimuli until a perceived US unpleasantness of 6 was achieved on a scale from 0 = “not painful at all” to 10 = “very painful”, with 4 indicating “just noticeable pain”. After calibration, the mean US intensities were 1.75 ± 2.14 mA (*M* ± SD).

### 2.3 Procedure

After arrival at the laboratory, participants were informed about the procedure of the study and signed informed consent. To measure incidental learning, participants were not informed about the upcoming memory task. Next, the EEG net and sensors for physiological recordings were applied, followed by individual calibrations of the pain‐threshold and the eye‐tracking system. The main experiment comprised 270 trials, which were divided into three conditions (see Figure 1), closely following the design of Rösler and Gamer (2019). Each trial started with a white fixation cross displayed for 4–6 s (ITI). Subsequently, a change in color of the fixation cross, either red, green, or yellow (for 2 s), signaled the upcoming condition. This was followed by the presentation of a neutral‐expression face for 6 s, after which the corresponding angry‐expression image was shown for 1 s. At the offset of the angry image, participants either received an inevitable US (inevitable threat condition; red cue), no US (safety condition; green cue), or could prevent the US by quickly pressing the space bar with their right hand during the angry facial expression period (avoidable threat condition; yellow cue). To ensure that threat avoidance was demanding and only successful in approximately 50% of the trials, the response time threshold was initially set to 250 ms for the first five trials and then dynamically adjusted to the median of the five most recent responses. This procedure resulted in an average of 47.8% (SD = 10.8%) shock trials in the avoidable threat condition. Crucially, all trials in the avoidable threat condition were included in the analysis, regardless of whether participants successfully avoided the threat or not. The trial type was randomized with the constraint that no more than three consecutive trials of the same condition occurred in succession. The total duration of the experiment was about 90 min. Participants were allowed to take self‐paced breaks after every 90 trials.

**FIGURE 1:**
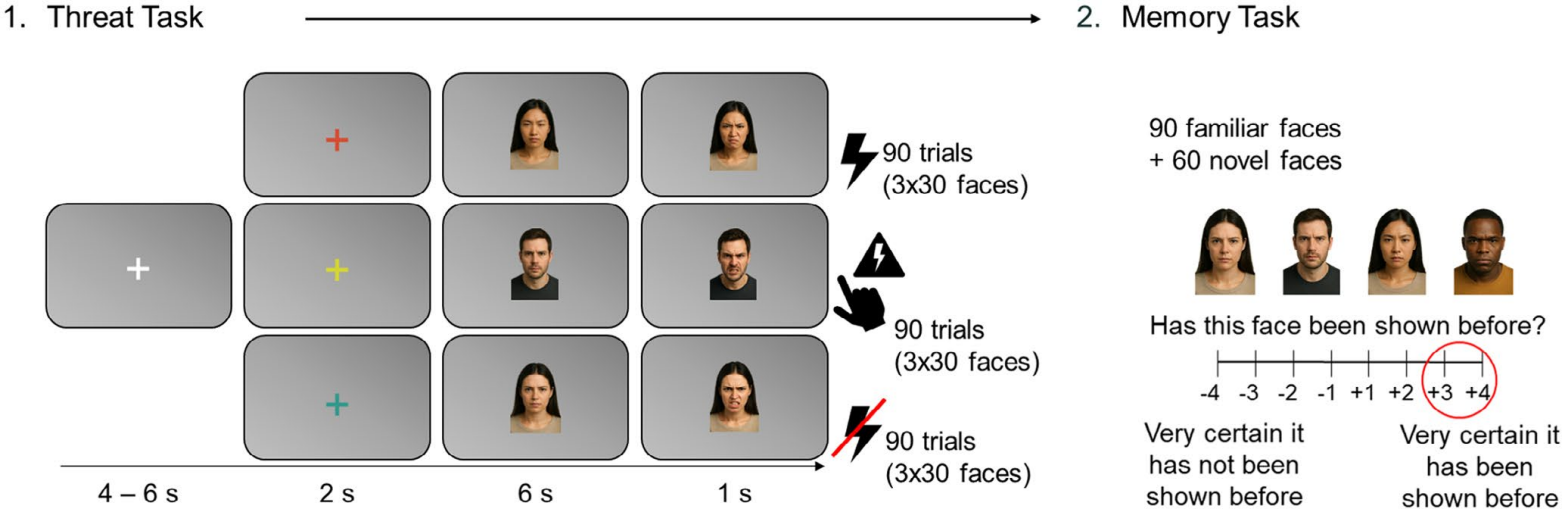
Design and trial structure of the main and memory task. Each trial began with a 4–6 s display of a white fixation cross (intertrial interval, ITI), followed by a 2 s color cue (red, green, or yellow) indicating the upcoming condition. A neutral face was then presented for 6 s before changing to an angry expression for 1 s. At the offset of the angry face, participants either (1) received a certain US (inevitable threat, red), (2) received no US (safety, green), or (3) had the opportunity to avoid the US by quickly pressing the space bar (avoidable threat, yellow). For each participant, 90 neutral face images were randomly selected and assigned to one of the three conditions. Each image was shown three times within the same condition, resulting in 90 trials per condition. During the memory task, the 90 previously seen faces and 60 novel faces were presented and categorized as remembered or forgotten based on participants' memory ratings. The faces shown here are illustrative examples and were not used in the original experiment.

Between the threat and memory tasks, participants spent approximately 20 min completing questionnaires and re‐checking EEG sensor impedances. This interval was chosen in line with previous studies (Leimeister et al. 2023; Wiemer et al. 2024, 2021) and served to allow for a brief period of memory consolidation (Dunsmoor et al. 2018), before participants viewed each facial identity again and were asked to indicate whether the given picture has been shown in the previous phase. The confidence in their judgment was rated on an eight‐point scale ranging from −4 (very certain it has not been shown before) to +4 (very certain it has been shown before), excluding zero. Faces were categorized as remembered or forgotten, depending on memory ratings: High confidence hits (+4 and +3) were classified as correctly remembered, while all other ratings were classified as forgotten (Leimeister et al. 2023; Wiemer et al. 2024, 2021). To assess the robustness of our findings, we repeated all analyses using a more liberal classification criterion, in which all hits greater than 0 were classified as correctly remembered and all other responses as forgotten. These analyses that yielded largely comparable results are reported in the Supporting Information.

### 2.4 Data Acquisition and Processing

#### 2.4.1 EEG

The EEG was continuously recorded using a 129‐electrodes net at a sampling rate of 500 Hz, referenced to Cz. The data were online filtered with a 100 Hz lowpass and a 50 Hz notch filter. All impedances were kept below 50 kΩ where possible. Offline processing was conducted in Matlab (The MathWorks Inc., version: 9.2.0, R2017a, Natick, Massachusetts) using the EMEGS (Electro Magnetic Encephalography) software (Peyk et al. 2011). First, epochs of −600 to 8000 ms relative to the trial onset were extracted from the continuous EEG. Data were then filtered with a 40 Hz low‐pass filter (23rd‐order Butterworth) and a 1 Hz high‐pass filter (1st‐order Butterworth). In the next step, the SCADS procedure (Junghöfer et al. 2000) was employed for artifact handling. Trials with artifacts were identified based on the distribution of statistical parameters, such as absolute value, standard deviation, and maximum differences, on trial and sensor level. Contaminated sensors were automatically identified and replaced using statistically weighted, spherical spline interpolated values. Trials with more than 20 out of 129 contaminated sensors were excluded from the analysis. On average, we excluded 30.7% ± 12.1% trials from the inevitable threat, 29.0% ± 12.5% trials from the avoidable threat, and 33.3% ± 12.3% trials from the safety condition. Artifact‐free trials were then submitted to a time‐frequency analysis. Morlet Wavelets were used with a Morlet coefficient of *m* = 10, applied to artifact‐free single trials across a frequency range from 2 to 40 Hz, followed by an averaging of the single trial time‐frequency representations by condition. The resulting native frequency resolution, based on a sampling rate of 500 Hz and 4300 samples per trial, was 0.1163 Hz. Baseline division was applied based on a time segment ranging from −400 to −240 ms. The alpha band was defined as the range between 9.1 and 12.6 Hz for the center frequencies of the Morlet wavelets. Consequently, we obtained a *σ*_*t*_ = 0.175 and *σ*_*f*_ = 0.960 for the lowest and *σ*_*t*_ = 0.126 and *σ*_*f*_ = 1.260 for the highest frequencies, respectively. We quantified changes in alpha power in response to the cues by averaging the data across nine sensors over the right parieto‐occipital region (EGI sensors 76, 82, 83, 84, 89, 90, 91, 95, 96; see Figure 3). This approach deviated from the preregistration, which specified Pz and its eight nearest neighbors, as the strongest grand‐average alpha suppression was lateralized toward the right hemisphere, consistent with our previous findings (Stegmann et al. 2024). Changes in alpha activity were then analyzed in three pre‐registered time windows: (1) 500–1000 ms following fixation cross onset, (2) 500–1000 ms following the neutral facial cue onset, and (3) 5000–6000 ms relative to the neutral facial cue onset.

#### 2.4.2 ECG

ECG was recorded at a sampling rate of 1000 Hz using three adhesive Ag/AgCl electrodes, placed underneath the right clavicle, as well as the left and right costal margin. Offline, ECG data were filtered using a 2 Hz high‐pass filter to remove slow signal drifts. Afterwards, R‐waves were detected from the ECG recordings in PeakMan 0.4.0 (https://github.com/dgromer/peakman) using a semi‐automatic method and manually edited in case of detection errors. R–R intervals were converted to HR in beats per minute. Changes in heart rate were analyzed in a pre‐registered time window of 5000–6000 ms following facial cue onset, immediately preceding the offset of the neutral face, relative to a baseline interval from −1000 to 0 ms before trial onset.

#### 2.4.3 Eye‐Tracking

Eye movements and pupil diameter were recorded with a Tobii Pro Nano eye‐tracker (Tobii Technology Inc., Tokyo, Japan) at a sampling rate of 60 Hz. Both eyes were recorded, and the mean *x*‐ and *y*‐coordinates on the screen, as well as the mean pupil size of both eyes were stored. Participants sat at a fixed viewing distance of 80 cm with unrestrained head movements. Eye‐tracking calibration was done using a nine‐point calibration procedure in Presentation before each of the three blocks. Fixations and saccades were detected using an event detection algorithm with a predefined minimal duration of 80 ms and maximal dispersion of 50 pixels (i.e., 0.9° of visual angle) per fixation. An iterative outlier detection algorithm was applied to ensure that participants fixated at the center of the screen at trial start (Rösler and Gamer 2019). Therefore, fixation locations during the last 300 ms before stimulus onset were considered as baseline for every trial. The smallest and the largest values were temporarily removed from the distribution of baseline coordinates and marked as outlier if they deviated more than 3 standard deviations from the mean baseline position of the remaining data. Trials with invalid baselines (i.e., substantial deviations from the initial gaze positions of the remaining data) were then excluded, and the procedure was repeated until no more baseline location was marked as outlier. Trials with baseline outliers or missing baseline position data (21.1%) as well as all subjects with more than 50% of missing or outlier baselines (*n* = 5) were excluded from all further eye‐tracking analyses. For the cleaned dataset, the *x*‐ and *y*‐coordinates of the baseline were then subtracted from the fixation coordinates during stimulus exploration. Then fixations were separated into bins of 1 s each for the 6 s picture‐viewing period. Similar to our previous research (Stegmann et al. 2024), we conducted additional analyses beyond the preregistered variables and calculated three different oculomotor metrics for the time window 5000–6000 ms relative to the neutral facial cue onset: the average distance of fixations from the center of the screen in pixels (center bias), the duration of individual fixations, and the number of fixations.

#### 2.4.4 Pupil Width

After extracting pupil diameter from the eye‐tracking data, we performed a linear interpolation on segments containing blinks and missing data. Following this, we applied a 2 Hz low‐pass filter and conducted a baseline correction by subtracting the mean of the −500 to 0 ms time window, relative to the trial onset, from each ensuing datapoint (Stegmann et al. 2024). For the statistical analysis, we calculated the average pupil diameter in three pre‐registered time windows: (1) 1000–2000 ms following fixation cross onset, (2) 1000–2000 ms following facial cue onset, and (3) 5000–6000 ms following facial cue onset, immediately preceding the offset of the neutral face.

### 2.5 Statistical Analysis

First, mean memory ratings and the resulting number of correctly remembered faces were compared between conditions using a repeated‐measures ANOVA with the within‐subject factors cue (3 levels: inevitable threat, avoidable threat, and safety). Then, all other dependent variables were analyzed using repeated‐measures ANOVAs with the within‐subject factors Cue (3 levels: inevitable threat, avoidable threat, and safety) and Memory (2 levels: forgotten vs. remembered). Degrees of freedom were adjusted according to Greenhouse–Geisser to compensate for potential violations of the sphericity assumption (Greenhouse and Geisser 1959). Post hoc *t*‐tests were performed to follow up on significant effects within the pre‐specified time windows mentioned above. All analyses were conducted in the R software environment (version 4.4.2.; R Development Core Team 2021), using the afex‐package for ANOVAs (Singmann et al. 2020; version 1.4‐1). Confidence intervals (95%) for partial eta‐squared ($\eta_{p}^{2}$) were calculated with the MBESS‐package (Kelley 2020; version 4.9.3). Statistical significance was determined using an alpha level of 0.05.

Additionally, we obtained converging evidence from Bayesian analyses. Bayesian ANOVAs were conducted using the *anovaBF* function from the *BayesFactor* package (Morey and Rouder 2026; version 0.9.12), with Jeffries priors and 100,000 Monte Carlo iterations (Rouder and Morey 2012). For repeated‐measures ANOVAs, Bayes Factors were computed for all combinations of main and interaction effects relative to a null model that included only the grand mean and participants' means as random intercepts. For main effects, Bayes factors (BF_10_) quantify evidence for the specified model over the null model. For interaction effects, Bayes factors (BF_int_) were obtained by comparing the full model, including the interaction term, to the corresponding model containing only main effects. Follow‐up *t*‐tests were conducted using the *ttestBF* function, which applies a Cauchy prior to the standardized effect size (Rouder and Morey 2012).

To identify predictors of flight response times on a trial‐by‐trial basis, we further computed a linear mixed model (LMM) including changes in alpha activity, heart rate, pupil dilation, distance of fixations from the center of the screen, and subsequent memory as fixed effects. The model also included participant‐specific random intercepts and random slopes for all fixed effects, including correlations among the random effects. As in previous studies (Merscher and Gamer 2023; Merscher et al. 2022; Rösler and Gamer 2019; Stegmann et al. 2024), we focused on the second half of the picture presentation, calculating the average responses on a trial‐wise basis in an interval between 5000 and 8000 ms relative to cue onset. All variables were *z*‐standardized prior to the analysis. The LMMs were performed in R, using the lmer‐package (Bates et al. 2015; version 1.1‐31) with a restricted maximum likelihood criterion. We obtained *p*‐values for each predictor from *t*‐tests using the Satterthwaite's approximation of degrees of freedom.

## 3 Results

### 3.1 Ratings

#### 3.1.1 Memory

In general, participants clearly distinguished between old faces (memory rating = 1.69 ± 0.93) and new faces (−2.31 ± 0.91), resulting in an average of 53.0% correctly remembered trials for inevitable threat, 53.3% for avoidable threat, and 53.7% for safety trials, whereas only 2.8% of new faces were incorrectly classified as remembered, *F*(2.16, 127.5) = 228.39, *p* < 0.001, $\eta_{p}^{2}$ = 0.79, CI_95_ = [0.74; 0.83], BF_10_ = 1.12 e+59, see Figure 2. However, there were no differences between the three threat task conditions in either memory ratings, *F*(1.54, 90.61) = 0.11, *p* = 0.840, $\eta_{p}^{2}$ < 0.01, CI_95_ = [0.00; 0.02], BF_10_ = 0.06, or the number of correctly remembered faces, *F*(1.71, 100.96) = 0.10, *p* = 0.878, $\eta_{p}^{2}$ < 0.01, CI_95_ = [0.00; 0.01], BF_10_ = 0.06.

**FIGURE 2:**
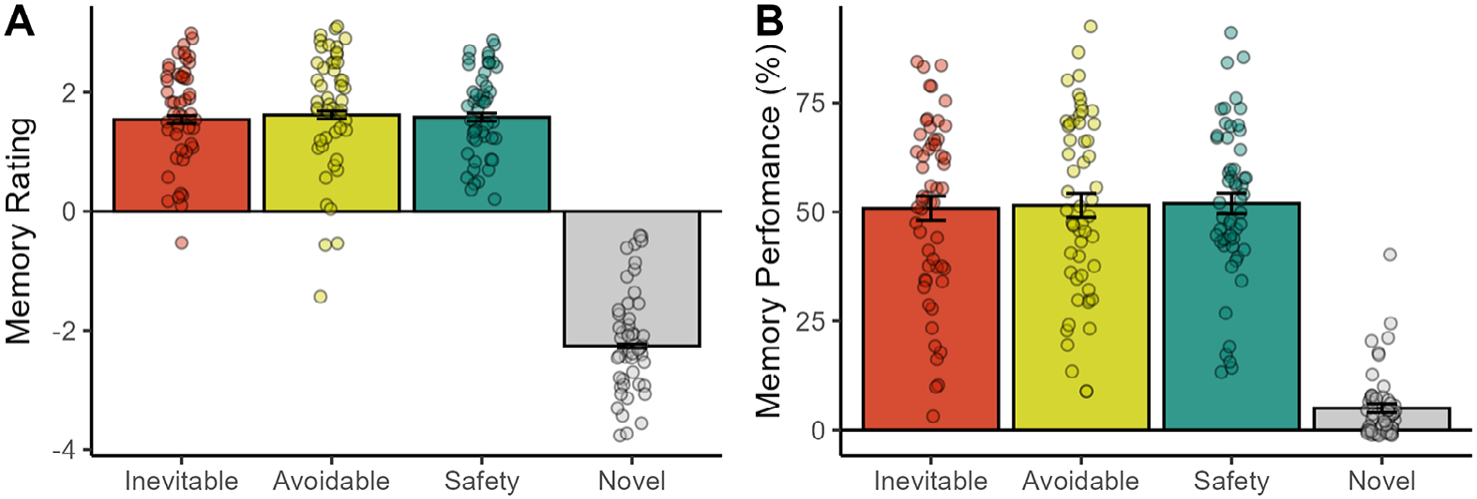
Memory ratings and performance across experimental conditions. (A) Mean memory ratings for previously encountered compared to novel faces. Each bar represents the group mean ± SEM, with individual participant data overlaid as scatter points. Higher values indicate stronger subjective memory. (B) Memory performance expressed as percentage of correctly recognized faces for each condition. In the novel condition, values reflect false positive rates. As in Panel A, bars depict group means ± SEM with individual data points shown. Participants demonstrated better memory for previously seen faces relative to novel faces.

### 3.2 Alpha Activity

Changes in alpha activity were analyzed in three pre‐registered time windows: (1) 500–1000 ms following the onset of the colored fixation cross, (2) 500–1000 ms following the neutral facial cue onset, and (3) 5000–6000 ms relative to the neutral facial cue onset (end of trial). These windows were selected based on our previous findings (Stegmann et al. 2024), which indicated that responses to the fixation cue and face onset likely reflect an orienting response, whereas sustained responses later in the trial reflect a more prolonged, freezing‐like response. While no explicit hypotheses were preregistered regarding differences between these time windows, we anticipated stronger threat‐related responses to stimulus onsets and increased motor‐preparation‐related responses toward the end of avoidable threat trials. EEG results are shown in Figure 3.

**FIGURE 3:**
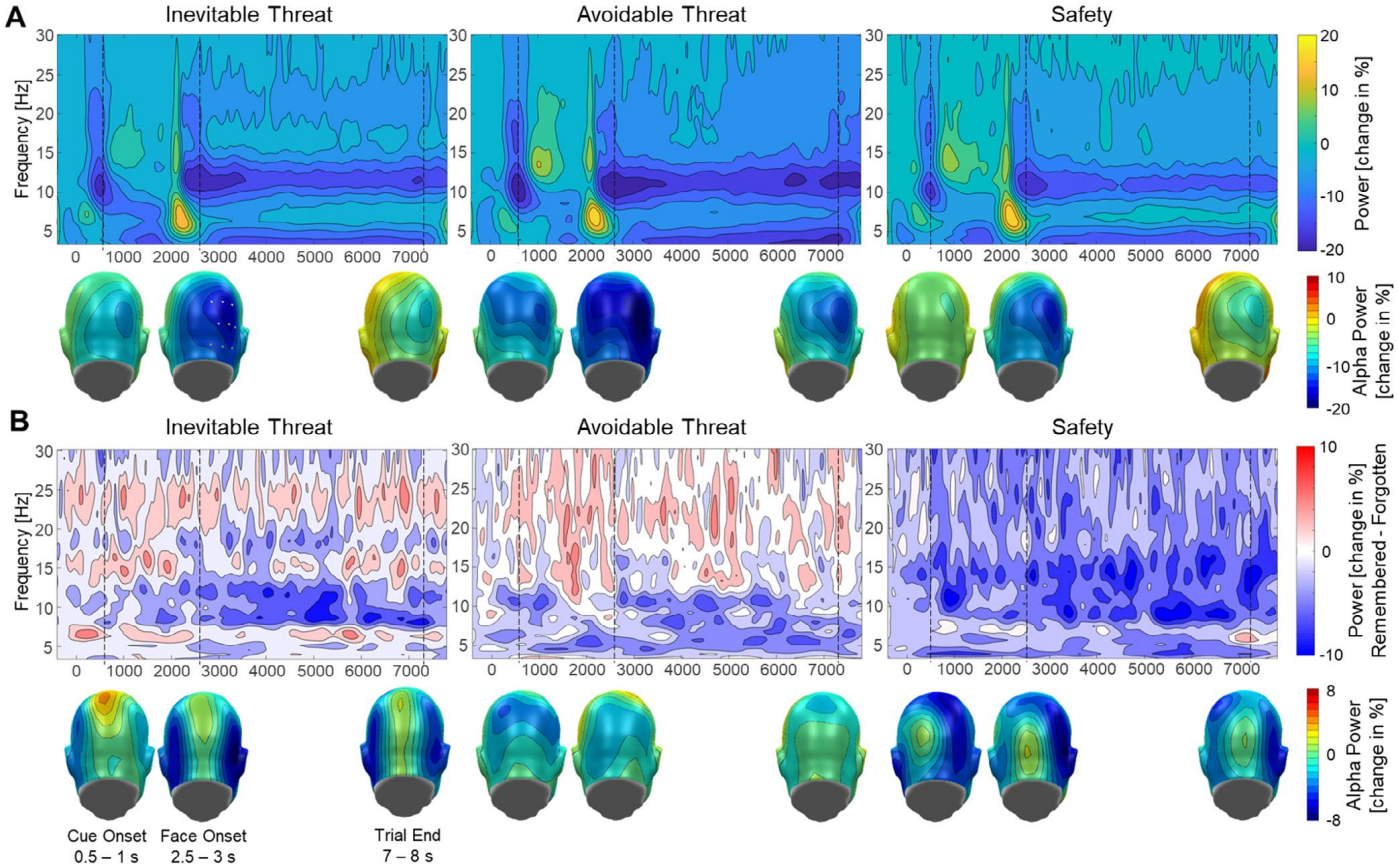
(A) Oscillatory activity in the different experimental conditions. Time–frequency representations and topographical scalp maps for the three preregistered time windows showing changes in neural (alpha) activity across the three experimental conditions. (B) Subsequent memory effect in oscillatory activity. Depicted are the differences between later remembered versus forgotten faces with darker blues indicating stronger neural (alpha) suppression for later remembered faces.

#### 3.2.1 Cue Onset

The Memory × Cue ANOVA for the fixation‐cross period revealed a significant main effect of Memory, *F*(1, 59) = 7.45, *p* = 0.008, $\eta_{p}^{2}$ = 0.11, CI_95_ = [0.01; 0.27], BF_10_ = 1.17, indicating stronger alpha suppression for subsequently remembered faces already during the presentation of the colored fixation cross signaling the trial type. In addition, there was a significant main effect of Cue, *F*(2, 118) = 8.90, *p* < 0.001, $\eta_{p}^{2}$ = 0.13, CI_95_ = [0.03; 0.24], BF_10_ = 545.39. Post hoc *t*‐tests showed stronger alpha suppression for avoidable threat, *t*(59) = 3.70, *p* < 0.001, *d* = 0.48, CI_95_ = [0.21; 0.74], BF_10_ = 52.04, and inevitable threat, *t*(59) = 3.47, *p* < 0.001, *d* = 0.45, CI_95_ = [0.18; 0.71], BF_10_ = 27.39, versus safety trials, whereas no difference emerged between avoidable and inevitable threat trials, *t*(59) = 0.91, *p* = 0.366, *d* = 0.12, CI_95_ = [−0.14; 0.37], BF_10_ = 0.21. The Memory × Cue interaction was not significant, *F*(2, 118) = 1.43, *p* = 0.244, $\eta_{p}^{2}$ = 0.02, CI_95_ = [0.00; 0.09], BF_int_ = 0.17.

#### 3.2.2 Face Onset

In the time window following the face onset, the ANOVA again revealed a significant main effect of Memory, *F*(1, 59) = 9.15, *p* = 0.004, $\eta_{p}^{2}$ = 0.13, CI_95_ = [0.02; 0.29], BF_10_ = 2.62, with stronger alpha suppression for subsequently remembered faces. The main effect of Cue was also significant, *F*(2, 118) = 3.18, *p* = 0.045, $\eta_{p}^{2}$ = 0.05, CI_95_ = [0.00; 0.14], BF_10_ = 1.27, but not the Memory × Cue interaction, *F*(1.75, 103.26) = 0.57, *p* = 0.545, $\eta_{p}^{2}$ < 0.01, CI_95_ = [0.00; 0.06], BF_int_ = 0.08. Post hoc *t*‐tests for the Cue effect revealed a significant difference between avoidable threat and safety trials, *t*(59) = 2.58, *p* = 0.012, *d* = 0.33, CI_95_ = [0.07; 0.59], BF_10_ = 2.93, but not between avoidable and inevitable threat trials, *t*(59) = 1.21, *p* = 0.231, *d* = 0.16, CI_95_ = [−0.10; 0.41], BF_10_ = 0.28, or threat and safety trials, *t*(59) = 1.31, *p* = 0.197, *d* = 0.17, CI_95_ = [−0.09; 0.42], BF_10_ = 0.32.

#### 3.2.3 Trial End

During the final second before the offset of the neutral face, the ANOVA again showed significant main effects of Memory, *F*(1, 59) = 9.21, *p* = 0.006, $\eta_{p}^{2}$ = 0.12, CI_95_ = [0.01; 0.28], BF_10_ = 1.89, and Cue, *F*(2, 118) = 9.58, *p* < 0.001, $\eta_{p}^{2}$ = 0.14, CI_95_ = [0.04; 0.25], BF_10_ = 1281.87. Post hoc *t*‐tests for the Cue effect revealed stronger alpha suppression during avoidable threat versus safety trials, *t*(59) = 4.44, *p* < 0.001, *d* = 0.57, CI_95_ = [0.30; 0.84], BF_10_ = 508.16, and avoidable versus inevitable threat trials, *t*(59) = 2.57, *p* = 0.013, *d* = 0.33, CI_95_ = [0.07; 0.59], BF_10_ = 2.88, whereas no difference was observed between safety and inevitable threat trials, *t*(59) = 1.62, *p* = 0.111, *d* = 0.21, CI_95_ = [−0.05; 0.46], BF_10_ = 0.48. The Memory × Cue interaction was again non‐significant, *F*(2, 118) = 1.69, *p* = 0.118, $\eta_{p}^{2}$ = 0.03, CI_95_ = [0.00; 0.10], BF_int_ = 0.20 suggesting generally stronger alpha suppression for subsequently remembered versus forgotten faces independent of the condition.

### 3.3 Heart Rate Responses

The Memory × Cue ANOVA for the pre‐registered time window of the final second before the facial stimulus offset revealed a significant main effect of Cue, *F*(1.49, 87.67) = 78.45, *p* < 0.001, $\eta_{p}^{2}$ = 0.57, CI_95_ = [0.45; 0.65], BF_10_ = 2.83 e+40. Post hoc *t*‐tests showed stronger cardiac deceleration for avoidable threat versus safety, *t*(59) = 10.06, *p* < 0.001, *d* = 1.30, CI_95_ = [0.96; 1.64], BF_10_ = 3.54 e+10, and inevitable threat trials, *t*(59) = 9.29, *p* < 0.001, *d* = 1.20, CI_95_ = [0.86; 1.53], BF_10_ = 2.11 e+10, but also for inevitable threat versus safety trials, *t*(59) = 5.88, *p* < 0.001, *d* = 0.76, CI_95_ = [0.47; 1.04], BF_10_ = 6.97 e+4. The absence of a significant main effect of Memory, *F*(1, 59) = 0.01, *p* = 0.942, $\eta_{p}^{2}$ < 0.01, CI_95_ = [0.00; 0.03], BF_10_ = 0.11, or Memory × Cue interaction, *F*(1.75, 103.10) = 0.08, *p* = 0.905, $\eta_{p}^{2}$ < 0.01, CI_95_ = [0.00; 0.02], BF_int_ = 0.05, suggests that changes in heart rate were not related to memory encoding (see Figure 4A).

**FIGURE 4:**
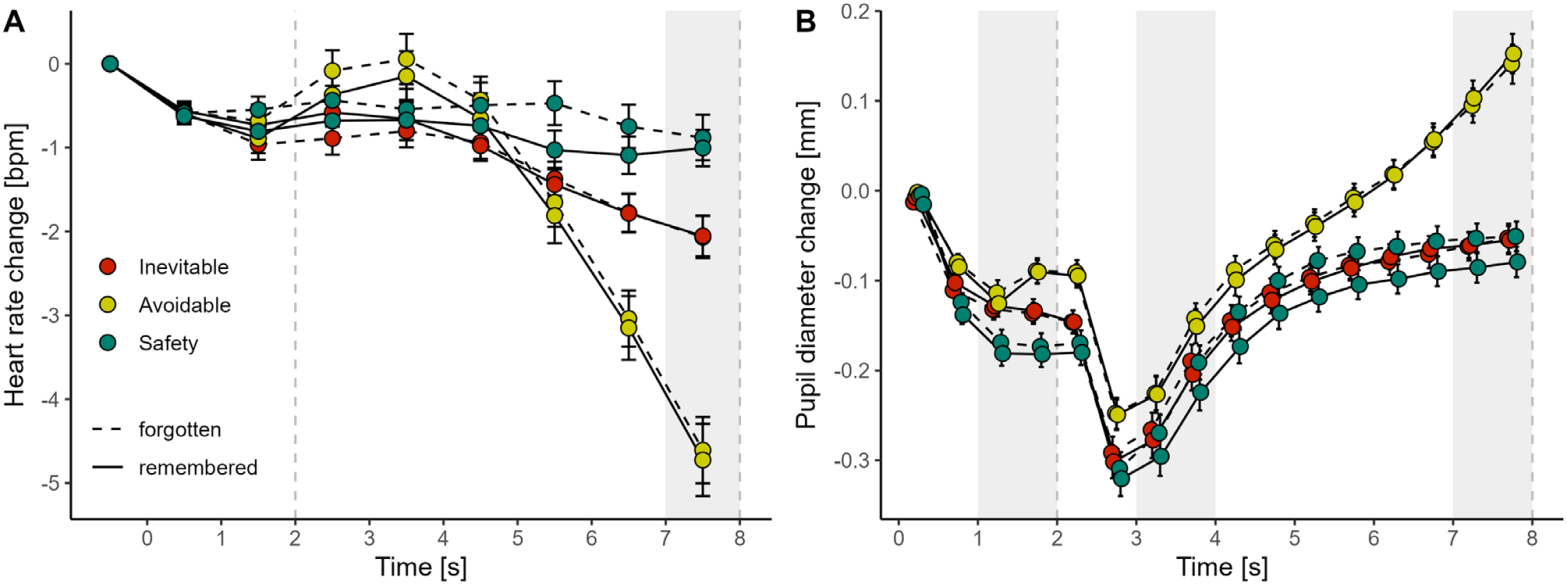
Autonomic responses as a function of memory outcome and cue condition. (A) Heart rate change (bpm) over time for inevitable threat (red), avoidable threat (yellow), and safety (green) trials, separated by subsequently remembered (solid lines) versus forgotten (dashed lines) faces. (B) Pupil diameter change (mm) over time for the same conditions. Error bars represent ±1 SEM with shaded areas indicating pre‐registered analysis windows. Vertical dashed lines represent the onset and offset of the neutral facial expressions, respectively. For illustrative purposes, changes in heart rate and pupil diameter were averaged into 1 s and 0.5 s bins, respectively.

### 3.4 Pupillary Responses

Parallel to the analysis of the alpha activity, we analyzed changes in pupil diameter in three pre‐registered time windows: (1) 1000–2000 ms following the onset of the colored fixation cross, (2) 1000–2000 ms following the neutral facial cue onset, and (3) 5000–6000 ms relative to the neutral facial cue onset (end of trial). Pupil results are shown in Figure 4B.

#### 3.4.1 Cue Onset

The Memory × Cue ANOVA for the fixation‐cross period revealed a significant main effect of Cue, *F*(2, 118) = 22.22, *p* < 0.001, $\eta_{p}^{2}$ = 0.27, CI_95_ = [0.14; 0.39], BF_10_ = 5.81 e+12. Post hoc *t*‐tests showed stronger pupil dilation for avoidable threat versus safety trials, *t*(59) = 6.77, *p* < 0.001, *d* = 0.87, CI_95_ = [0.57; 1.17], BF_10_ = 1.80 e+6, avoidable versus inevitable threat trials, *t*(59) = 2.53, *p* = 0.014, *d* = 0.33, CI_95_ = [0.07; 0.58], BF_10_ = 2.60, and inevitable threat versus safety trials, *t*(59) = 4.49, *p* < 0.001, *d* = 0.58, CI_95_ = [0.30; 0.85], BF_10_ = 602.51. In addition, the Memory × Cue ANOVA was significant, *F*(2, 118) = 3.26, *p* = 0.042, $\eta_{p}^{2}$ = 0.05, CI_95_ = [0.00; 0.14], BF_int_ = 0.28 e+10. Here, post hoc *t*‐tests showed a significantly reduced pupil dilation for later remembered versus forgotten faces only in the inevitable threat condition, *t*(59) = 2.40, *p* = 0.019, *d* = 0.31, CI_95_ = [0.05; 0.57], BF_10_ = 2.00, but not in the avoidable threat, *t*(59) = 1.35, *p* = 0.183, *d* = 0.17, CI_95_ = [−0.08; 0.43], BF_10_ = 0.11, or safety conditions, *t*(59) = 0.26, *p* = 0.799, *d* = 0.03, CI_95_ = [−0.29; 0.22], BF_10_ = 0.14. The main effect of memory was not significant, *F*(1, 59) = 0.70, *p* = 0.405, $\eta_{p}^{2}$ < 0.01, CI_95_ = [0.00; 0.11], BF_10_ = 0.13.

#### 3.4.2 Face Onset

In the window following face onset, the ANOVA revealed only a significant main effect of Cue, *F*(2, 118) = 19.91, *p* < 0.001, $\eta_{p}^{2}$ = 0.25, CI_95_ = [0.12; 0.37], BF_10_ = 1.28 e+10, but no main effect of Memory, *F*(1, 59) = 2.33, *p* = 0.132, $\eta_{p}^{2}$ = 0.04, CI_95_ = [0.00; 0.17], BF_10_ = 0.21, and only a marginally significant Memory × Cue interaction, *F*(2, 118) = 2.60, *p* = 0.078, $\eta_{p}^{2}$ = 0.04, CI_95_ = [0.00; 0.12], BF_int_ = 0.22. Post hoc *t*‐tests for the Cue effect revealed a stronger pupil dilations for avoidable threat versus safety trials, *t*(59) = 6.43, *p* < 0.001, *d* = 0.83, CI_95_ = [0.53; 1.12], BF_10_ = 5.15 e+5, avoidable versus inevitable threat trials, *t*(59) = 5.30, *p* < 0.001, *d* = 0.68, CI_95_ = [0.40; 0.96], BF_10_ = 9.00 e+3, and inevitable threat versus safety trials, *t*(59) = 2.10, *p* = 0.040, *d* = 0.27, CI_95_ = [0.01; 0.53], BF_10_ = 1.08.

#### 3.4.3 Trial End

During the final second before the offset of the neutral face, the ANOVA again showed a significant main effect Cue, *F*(1.44, 85.24) = 65.50, *p* < 0.001, $\eta_{p}^{2}$ = 0.53, CI_95_ = [0.40; 0.61], BF_10_ = 1.10 e+34, with stronger pupil dilation for avoidable threat versus safety trials, *t*(59) = 9.55, *p* < 0.001, *d* = 1.23, CI_95_ = [0.89; 1.57], BF_10_ = 5.51 e+10, and avoidable versus inevitable threat trials, *t*(59) = 8.85, *p* < 0.001, *d* = 1.14, CI_95_ = [0.81; 1.47], BF_10_ = 4.13 e+9, but not for inevitable threat versus safety trials, *t*(59) = 1.22, *p* = 0.226, *d* = 0.16, CI_95_ = [−0.10; 0.41], BF_10_ = 0.29. In addition, there was a significant main effect of Memory, *F*(1, 59) = 21.32, *p* < 0.001, $\eta_{p}^{2}$ = 0.27, CI_95_ = [0.09; 0.43], BF_10_ = 1.83. Against expectations, however, pupil dilation was reduced for subsequently remembered versus forgotten faces. The Memory × Cue interaction was again non‐significant, *F*(2, 118) = 2.20, *p* = 0.116, $\eta_{p}^{2}$ = 0.04, CI_95_ = [0.00; 0.11], BF_10_ = 0.14.

### 3.5 Oculomotor Responses

#### 3.5.1 Center Bias

The Memory × Cue ANOVA for gaze distance to the center of the screen during the final second of the trial revealed a main effect of Cue, *F*(2, 108) = 12.17, *p* < 0.001, $\eta_{p}^{2}$ = 0.19, CI_95_ = [0.06; 0.30], BF_10_ = 2.22 e+3, but no main effect of Memory, *F*(1, 54) = 0.29, *p* = 0.594, $\eta_{p}^{2}$ < 0.01, CI_95_ = [0.00; 0.10], BF_10_ = 0.13, and no Memory × Cue interaction, *F*(1.79, 96.71) = 0.42, *p* = 0.636, $\eta_{p}^{2}$ < 0.01, CI_95_ = [0.00; 0.05], BF_10_ = 0.10 (see Figure 5A). Post hoc *t*‐tests indicated a stronger center bias for avoidable threat compared to safety, *t*(54) = 2.53, *p* < 0.001, *d* = 0.63, CI_95_ = [0.34; 0.92], BF_10_ = 940.85, and inevitable threat trials, *t*(54) = 2.73, *p* = 0.009, *d* = 0.37, CI_95_ = [0.09; 0.64], BF_10_ = 4.15, as well as for safety compared to inevitable threat trials, *t*(54) = 2.27, *p* = 0.027, *d* = 0.31, CI_95_ = [0.03; 0.58], BF_10_ = 1.56. To exploratorily examine potential memory effects, we re‐ran the ANOVA on gaze distance averaged across the entire trial (but excluding the final second). Importantly, these gaze analyses were guided by visual inspection of the data and were not preregistered. As these decisions were made post hoc, the corresponding results should be interpreted with caution and considered preliminary. This analysis revealed an additional significant main effect of Memory, *F*(1, 54) = 5.09, *p* = 0.028, $\eta_{p}^{2}$ = 0.09, CI_95_ = [0.00; 0.24], BF_10_ = 0.82, indicating a weaker center bias for subsequently remembered versus forgotten faces, while the interaction remained non‐significant, *F*(2, 108) = 0.79, *p* = 0.457, $\eta_{p}^{2}$ = 0.01, CI_95_ = [0.00; 0.07], BF_10_ = 0.10.

**FIGURE 5:**
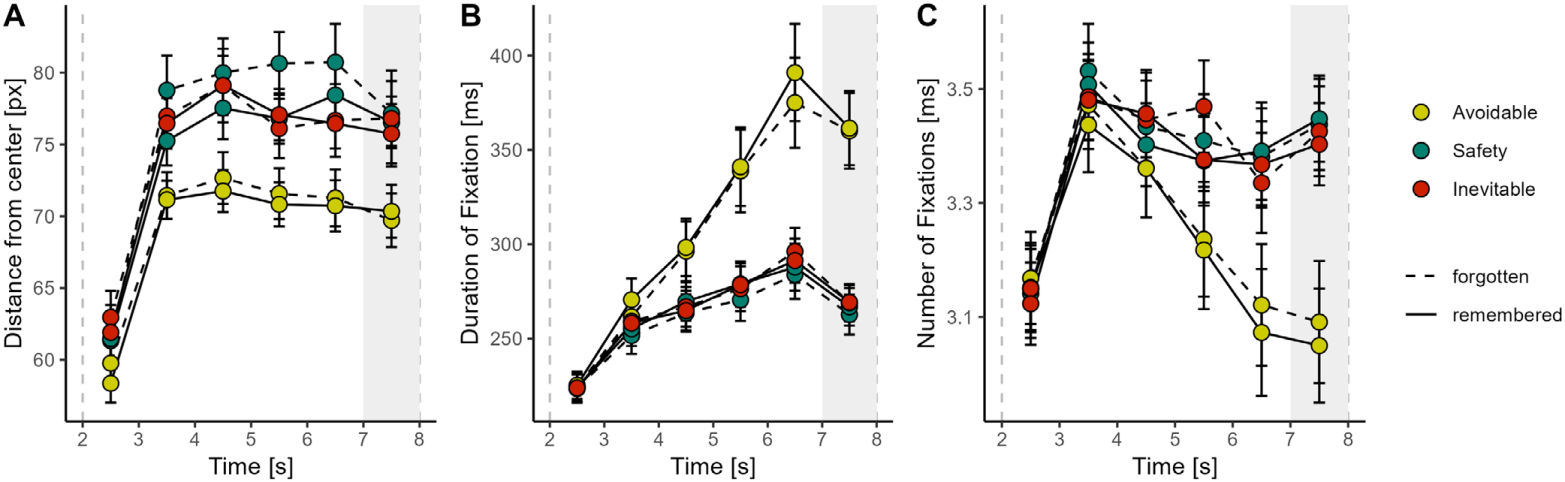
Oculomotor responses as a function of memory outcome and cue condition. (A) Distance from the center [in pixels] over time for inevitable threat (red), avoidable threat (yellow), and safety (green) trials, separated by subsequently remembered (solid lines) versus forgotten (dashed lines) faces. (B) Duration and (C) number of fixations over time for the same conditions. Error bars represent ±1 SEM with shaded areas indicating the preregistered analysis windows. Vertical dashed lines represent the onset and offset of the neutral facial expressions, respectively. For illustrative purposes, indices of oculomotor responses were averaged into 1 s bins.

#### 3.5.2 Duration of Fixations

A similar pattern was observed for fixation duration. The ANOVA for the final second of the trial revealed a main effect of Cue, *F*(1.12, 60.60) = 55.55, *p* < 0.001, $\eta_{p}^{2}$ = 0.51, CI_95_ = [0.37; 0.60], BF_10_ = 7.37 e+31, but no main effect of Memory, *F*(1, 54) < 0.01, *p* = 0.962, $\eta_{p}^{2}$ < 0.01, CI_95_ = [0.00; 0.01], BF_10_ = 0.12, and no Memory × Cue interaction, *F*(2, 108) = 0.17, *p* = 0.847, $\eta_{p}^{2}$ < 0.01, CI_95_ = [0.00; 0.03], BF_int_ = 0.06 (see Figure 5B). Post hoc *t*‐tests showed longer fixations for avoidable threat compared to safety, *t*(54) = 7.36, *p* < 0.001, *d* = 1.06, CI_95_ = [0.72; 1.39], BF_10_ = 6.02 e+7, and inevitable threat trials, *t*(54) = 7.36, *p* < 0.001, *d* = 0.99, CI_95_ = [0.67; 1.31], BF_10_ = 1.04 e+6, whereas no difference emerged between safety and inevitable threat trials, *t*(54) = 0.74, *p* = 0.464, *d* = 0.10, CI_95_ = [−0.17; 0.36], BF_10_ = 0.19. The exploratory full‐trial analysis (excluding the final second) revealed a main effect of Memory, *F*(1, 54) = 6.93, *p* = 0.011, $\eta_{p}^{2}$ = 0.11, CI_95_ = [0.01; 0.28], BF_10_ = 0.41, with shorter fixations for subsequently remembered versus forgotten faces. The interaction was again non‐significant, *F*(2,108) = 0.57, *p* = 0.569, $\eta_{p}^{2}$ = 0.01, CI_95_ = [0.00; 0.06], BF_int_ = 0.07.

#### 3.5.3 Number of Fixations

Likewise, the ANOVA for the number of fixations during the final second revealed a main effect of Cue, *F*(1.41, 75.88) = 40.47, *p* < 0.001, $\eta_{p}^{2}$ = 0.43, CI_95_ = [0.28; 0.53], BF_10_ = 7.36 e+31, but no main effect of Memory, *F*(1, 54) = 0.07, *p* = 0.895, $\eta_{p}^{2}$ < 0.01, CI_95_ = [0.00; 0.07], BF_10_ = 0.12, and no Memory × Cue interaction, *F*(2, 108) = 1.66, *p* = 0.195, $\eta_{p}^{2}$ = 0.03, CI_95_ = [0.00; 0.10], BF_int_ = 0.06 (see Figure 5C). Post hoc *t*‐tests showed fewer fixations for avoidable threat compared to safety, *t*(54) = 7.08, *p* < 0.001, *d* = 0.96, CI_95_ = [0.63; 1.27], BF_10_ = 3.88 e+6, and inevitable threat trials, *t*(54) = 6.43, *p* < 0.001, *d* = 0.87, CI_95_ = [0.55; 1.17], BF_10_ = 3.83 e+5, with no difference between safety and inevitable threat trials, *t*(54) = 1.00, *p* = 0.319, *d* = 0.14, CI_95_ = [−0.13; 0.40], BF_10_ = 0.24. Exploratory full‐trial analysis (excluding the final second) again revealed a main effect of Memory, *F*(1, 54) = 9.21, *p* = 0.004, $\eta_{p}^{2}$ = 0.15, CI_95_ = [0.02; 0.31], BF_10_ = 1.84, indicating more fixations for subsequently remembered versus forgotten faces, while the interaction was non‐significant, *F*(2,108) = 2.37, *p* = 0.098, $\eta_{p}^{2}$ = 0.04, CI_95_ = [0.00; 0.13], BF_int_ = 0.18.

In summary, the results for indices of oculomotor responses revealed a robust pattern of reduced eye‐movements during the end of trial, recently described as “freezing of gaze”, which did not relate to memory encoding. However, exploratory analysis for the entire interval suggested that increased visual exploration as indicated by a weaker center bias along with shorter, but more fixations was associated with better memory encoding.

### 3.6 Predictors of Avoidance Response Latencies

In avoidable threat trials, the participants' mean response time was 252.00 ms (SD = 88.9 ms). The linear mixed model revealed that faster responses were associated with an increased suppression of alpha activity, *t*(48.46) = 2.38, *p* = 0.022, *β* = 0.040, SE = 0.014, and heart rate deceleration, *t*(53.70) = 3.36, *p* = 0.002, *β* = 0.075, SE = 0.022. In contrast, the distance of fixations from the center of the screen, *t*(36.71) = 0.20, *p* = 0.843, *β* = 0.005, SE = 0.023, memory, *t*(52.82) = 1.33, *p* = 0.191, *β* = −0.052, SE = 0.039, and pupil dilation, *t*(43.61) = 1.42, *p* = 0.163, *β* = −0.029, SE = 0.020 did not prove to be a significant predictor of response times.

## 4 Discussion

In the present study, we investigated the oculomotor, autonomic, and electrocortical correlates of memory formation during states of attentive immobility. To this end, we assessed memory performance following a well‐established threat paradigm comparing the anticipation of avoidable, inevitable, and no threat (Merscher and Gamer 2023; Merscher et al. 2022; Rösler and Gamer 2019; Stegmann et al. 2024), while recording electroencephalographic, electrocardiographic, pupillometric, and oculomotor responses. We specifically tested whether increased suppression of alpha activity, cardiac deceleration, and pupil dilation during the anticipation of avoidable threat would translate into enhanced memory performance for stimuli presented during this period.

Consistent with our hypotheses, the anticipation of avoidable threat elicited markers of attentive immobility, reflected in posterior alpha suppression, heart rate deceleration, and pupil dilation. In addition, oculomotor indices revealed reduced eye movements toward the end of the trial, consistent with the “freezing of gaze” response described by Rösler and Gamer (2019). This pattern of responses corroborates the notion that attentive immobility is an active state, characterized by enhanced sensory intake and motor system activation with the ultimate goal to prepare for upcoming fight‐or‐flight responses (Roelofs 2017; Roelofs and Dayan 2022), probably driven by motivational relevance (Teigeler et al. 2025). Consistently, we were able to replicate our findings of more pronounced parieto‐occipital alpha suppression during avoidable threat trials compared with inevitable threat or safety trials (Stegmann et al. 2024). In line with previous research, reduced alpha power reflects enhanced processing of sensory information and increased cortical excitability (Foxe and Snyder 2011; Klimesch et al. 2007). Alpha oscillations are thought to play a central role in attentional selection and suppression, allowing the brain to prioritize relevant stimuli while filtering out distractions (Kelly et al. 2006; Klimesch 2012). Importantly, parieto‐occipital alpha activity is sensitive to the affective and motivational significance of visual stimuli, with stronger suppression observed for aversive or conditioned threat cues compared with neutral or safe stimuli (Bacigalupo and Luck 2022; Panitz et al. 2019; Pirazzini et al. 2023; Starita et al. 2023), suggesting that alpha suppression supports attentive immobility by engaging neural mechanisms that optimize the processing of threat‐relevant information (Lang and Bradley 2010).

In line with the notion that reduced alpha activity facilitates the allocation of attentional resources toward incoming information, we assumed that states of attentive immobility promote the consolidation of such information into long‐term memory. Supporting this view, studies comparing subsequently remembered versus forgotten items reported accumulating evidence for decreased alpha power during encoding predicting successful memory formation (Hanslmayr and Staudigl 2014). Likewise, pupil dilation is widely used as an index of autonomic arousal and locus coeruleus–noradrenergic activity (Gilzenrat et al. 2010; Korn and Bach 2016), both of which are known to modulate memory‐related neural plasticity (Hansen and Manahan‐Vaughan 2015). Larger pupil responses during encoding have repeatedly been linked to stronger memory traces (Leimeister et al. 2023; Pitem and Mama 2025; Wiemer et al. 2021), presumably reflecting heightened motivational significance or increased cognitive effort directed toward the stimulus. Although we observed increased pupil dilation and suppression of alpha activity in the avoidable threat condition, we found no evidence for enhanced memory performance. In fact, faces were remembered equally well across conditions. However, analyses of subsequent memory effects revealed distinct neurophysiological and oculomotor mechanisms related to memory encoding during the threat task. As expected, alpha suppression was stronger for later remembered than for forgotten faces. Yet, a closer inspection of the topographies and temporal dynamics showed that the strongest differences emerged over temporal‐occipital regions and during the middle phase of face presentation, rather than in the two preregistered time windows at face onset and trial end. Such a divergence in alpha dynamics may have two key implications. First, suppression of posterior alpha associated with attentional engagement may recruit neural processes that are partially distinct from those supporting successful memory encoding (Hanslmayr and Staudigl 2014). Second, while alpha suppression is commonly linked to enhanced sensory selection and prioritization, such attentional facilitation does not necessarily translate into improved memory encoding. Rather than reflecting a generalized state of heightened attentional processing, attentive immobility may instead support monitoring and action‐preparation mechanisms that are specifically tuned to immediate, behaviorally relevant demands.

This interpretation is further supported by the results of the autonomic measures. Similar to posterior alpha suppression, cardiac deceleration in response to external stimuli has been associated with *orienting* and enhanced sensory processing (Bradley 2009; Bradley et al. 2012; Martín et al. 2025). Previous studies have reported greater heart rate deceleration for subsequently remembered compared to forgotten pictures (Abercrombie et al. 2008) and words (Buchanan et al. 2006). In our study, although we observed cardiac deceleration during the anticipation period of both avoidable and inevitable threats, this did not translate into enhanced memory performance. Across all conditions, there was no evidence of a subsequent memory effect in heart rate. These results parallel our findings for alpha activity, suggesting that heart rate modulations primarily reflected the immediate demands of the task rather than contributing to episodic memory consolidation. Although we observed a subsequent memory effect for pupil dilation, it was in the opposite direction of what is typically reported. Whereas some studies find greater pupil dilation for later remembered compared to forgotten items (Leimeister et al. 2023; Pitem and Mama 2025; Wiemer et al. 2021), some previous investigations have also reported the reverse pattern (Lloyd et al. 2025; Naber et al. 2013; Pilarczyk et al. 2022; Wetzel et al. 2020). One plausible explanation is that arousal in our task was tightly coupled to response preparation rather than memory encoding. Higher arousal may have facilitated rapid detection and reaction to the facial expression change, thereby prioritizing task performance at the expense of deeper encoding of the faces themselves. Conversely, lower arousal may have allowed participants to disengage from immediate task demands and allocate more resources toward successful memory formation.

Our findings raise important questions about what drives memory for incidental stimulation under threat. Momentary fluctuations in attention may have contributed to memory formation, as indicated by the observation that alpha activity related to later memory differences was evident even during the presentation of the colored fixation cross, before any face was displayed. Additionally, other factors not directly related to attention or threat may influence memory outcomes. For example, since faces were randomly assigned to conditions, some stimuli may have been more memorable due to intrinsic properties such as perceived (un)trustworthiness or attractiveness (Lin et al. 2020; Rule et al. 2012; Weymar et al. 2019). These influences are likely idiosyncratic and difficult to control experimentally, highlighting that incidental memory under threat is probably shaped by a combination of state‐dependent attentional processes and stimulus‐specific characteristics.

Finally, although we did not preregister analyses of the oculomotor indices, as we did not expect substantial variations in visual exploration of faces compared to previously used landscape pictures (Rösler and Gamer 2019; Stegmann et al. 2024), these measures provided additional insight into the relationship between attentive immobility and memory. Across conditions, increased visual exploration of the faces was associated with better subsequent memory performance, consistent with previous findings showing a similar relationship during the observation and encoding of scenes (Fehlmann et al. 2020). Interestingly, this effect contrasted with the avoidable‐threat condition, in which participants exhibited reduced visual exploration. These findings further suggest that preparatory mechanisms associated with attentive immobility are not directly linked to the processes supporting memory encoding.

Taken together, the present study revealed a specific pattern of responses associated with memory encoding. Later‐remembered faces were characterized by stronger alpha suppression over temporo‐occipital regions, increased visual exploration, and reduced pupil dilation compared to later‐forgotten faces; effects that were independent of the condition in which the faces were presented. During avoidable‐threat conditions, however, attentive immobility was accompanied by increased alpha suppression, but increased pupil dilation and reduced visual exploration, suggesting that these mechanisms may counteract one another, potentially resulting in a zero net benefit for memory. On the other hand, it is likely that attentive immobility does not reflect a generalized state of heightened sensory intake; rather, it appears optimized for motor preparation and monitoring of behaviorally relevant task demands. Importantly, facial details themselves were not task‐relevant, as participants needed only to detect the change from a neutral to an angry expression. This aligns with the arousal‐biased competition (ABC) theory (Mather and Sutherland 2011), which posits that heightened arousal does not universally enhance memory; instead, an item's priority determines whether arousal facilitates or impairs its perception and encoding. In this framework, already salient stimuli “win” and receive further processing, whereas lower‐priority information is suppressed. In the current study, the neutral faces represented low‐priority stimuli, while the transition to an angry expression constituted the task‐relevant, high‐priority event. Accordingly, future studies should explore whether effects on memory performance emerge when task conditions are not explicitly signaled by a colored cue, but instead participants must learn the association between each facial identity and its corresponding condition. From a broader perspective, the absence of condition‐specific memory effects in the present study may be informative in its own right. Although states of heightened vigilance and defensive engagement are often assumed to modulate cognitive processing, our findings suggest that a state of attentive immobility does not necessarily translate into measurable changes in memory encoding. One possible interpretation is that such states primarily recalibrate action readiness and perceptual priorities without substantially altering downstream mnemonic processes. In this sense, attentive immobility may reflect a functional mode optimized for immediate situational demands rather than for the durable encoding of information. Importantly, this interpretation is consistent with our previous work, in which we demonstrated that threat intensity, avoidability, and motivational factors are tightly intertwined and jointly shape physiological and behavioral responses (Teigeler et al. 2025). In that context, both defensive reactions and motor preparation appeared to be driven by motivational relevance, which increases with perceived imminence, potential harm, and the possibility of escape. The present findings extend this perspective by suggesting that, while such states robustly influence action‐oriented systems, their impact on memory encoding may be more limited or context‐dependent.

While the present study provides novel insights into the neurophysiological and oculomotor mechanisms underlying memory encoding under avoidable threat, several limitations should be acknowledged. First, memory recall was assessed on the same day, following only a brief interval spent completing questionnaires between the threat and memory tasks. Although this interval was intended to reduce ongoing mental occupation with the stimuli from the main task, it did not allow for overnight consolidation, which is critical for stabilizing memory traces into long‐term memory (Gais et al. 2006; Talamini et al. 2008). This is particularly relevant given evidence that items encoded under heightened arousal are sometimes recalled less effectively immediately after learning, yet show enhanced retention following overnight consolidation (LaBar and Phelps 1998; Pierce and Kensinger 2011). Consequently, future studies should consider implementing a 2‐day paradigm, separating the main task and the memory assessment to allow for overnight consolidation. Second, similar to previous studies investigating subsequent memory effects for faces learned under threat (Leimeister et al. 2023; Wiemer et al. 2024, 2021), we classified items with memory confidence ratings of +3 and +4 as remembered, and those with ratings of +2 or lower as forgotten. While this dichotomization introduces somewhat arbitrary boundaries, it allows for a clear separation between high‐confidence and low‐confidence memory decisions, which facilitates the detection of robust subsequent memory effects. In addition, it produced roughly equal numbers of remembered and forgotten items, optimizing the statistical power of our analyses. Importantly, repeating all analyses using a simpler positive‐versus‐negative criterion yielded virtually identical results (see Supporting Information). Another limitation of the present study concerns the relatively long duration of the experimental paradigm. Although a high number of trials per condition was necessary to obtain reliable estimates of physiological responses and subsequent memory performance at the item level, extended task exposure may introduce habituation, fatigue, or fluctuations in engagement. Indeed, supplementary analyses revealed some attenuation of response magnitudes during the final third of the experiment, consistent with reduced arousal over time. While the critical differences between conditions remained largely intact, these findings suggest that prolonged paradigms may challenge the stability of defensive responding.

In summary, our findings demonstrate that anticipation of avoidable threats elicits a distinctive pattern of physiological and oculomotor responses, consisting of heart rate bradycardia, centralized gaze, suppressed visual alpha activity, and increased sympathetic arousal that reflects a state of attentive immobility. This state appears to optimize perceptual processing and readiness for action, supporting adaptive defensive behavior. Crucially, however, it did not directly facilitate memory encoding. Instead, subsequent memory effects were linked to reduced pupil dilation and decreased alpha activity over occipitotemporal regions, independent of threat condition. Together, these results indicate that attentive immobility during avoidable threat prioritizes immediate behavioral demands rather than facilitating long‐term memory formation, highlighting a dissociation between threat‐related attention and memory processes.

## Author Contributions

**Yannik Stegmann:** conceptualization, methodology, data curation, formal analysis, visualization, writing – original draft, investigation. **Matthias Gamer:** supervision, resources, project administration, writing – review and editing, funding acquisition.

## Conflicts of Interest

The authors declare no conflicts of interest.

## Supporting information


**Figure S1:** Heart rate changes (bpm) over time for inevitable (red), avoidable threat (yellow), and safety (green) trials, separated by subsequently remembered (solid lines) versus forgotten (dashed lines) faces and bins corresponding to thirds of the experiment (first, second, and third third of the experiment). Error bars represent ±1 SEM with shaded areas indicating pre‐registered analysis windows. Vertical dashed lines represent the onset and offset of the neutral facial expressions, respectively.
**Figure S2:** Changes in pupil diameter over time for inevitable (red), avoidable threat (yellow), and safety (green) trials, separated by subsequently remembered (solid lines) versus forgotten (dashed lines) faces and bins corresponding to thirds of the experiment (first, second, and third of the experiment). Error bars represent ±1 SEM with shaded areas indicating pre‐registered analysis windows. Vertical dashed lines represent the onset and offset of the neutral facial expressions, respectively.
**Figure S3:** Oculomotor responses as a function of memory outcome, cue condition, and third of experiment. (A) Distance from the center [in pixels] over time for inevitable (red), avoidable threat (yellow), and safety (green) trials, separated by subsequently remembered (solid lines) versus forgotten (dashed lines) faces. (B) Duration and (C) number of fixations over time for the same conditions. Error bars represent ±1 SEM with shaded areas indicating the analysis windows. Vertical dashed lines represent the onset and offset of the neutral facial expressions, respectively.
**Table S1:** Trials (*M ±* SD) available for analyses using the more liberal memory criterion.
**Figure S4:** Memory performance across experimental conditions expressed as percentage of correctly recognized faces for each condition. Bars depict means ± SEM with individual data points shown. Participants demonstrated better memory for previously seen faces relative to novel faces.
**Figure S5:** Changes in alpha activity as a function of memory outcome and cue condition over time for inevitable threat (red), avoidable threat (yellow), and safety (green) trials, separated by subsequently remembered (solid lines) versus forgotten (dashed lines) faces. Error bars represent ±1 SEM with shaded areas indicating pre‐registered analysis windows. Vertical dashed lines represent the onset and offset of the neutral facial expressions, respectively.
**Figure S6:** Autonomic responses as a function of memory outcome and cue condition. (A) Heart rate change (bpm) over time for inevitable threat (red), avoidable threat (yellow), and safety (green) trials, separated by subsequently remembered (solid lines) versus forgotten (dashed lines) faces. (B) Pupil diameter change (mm) over time for the same conditions. Error bars represent ±1 SEM with shaded areas indicating pre‐registered analysis windows. Vertical dashed lines represent the onset and offset of the neutral facial expressions, respectively.
**Figure S7:** Oculomotor responses as a function of memory outcome and cue condition. (A) Distance from the center [in pixels] over time for inevitable threat (red), avoidable threat (yellow), and safety (green) trials, separated by subsequently remembered (solid lines) versus forgotten (dashed lines) faces. (B) Duration and (C) number of fixations over time for the same conditions. Error bars represent ±1 SEM with shaded areas indicating the analysis windows. Vertical dashed lines represent the onset and offset of the neutral facial expressions, respectively.

## Data Availability

The data that support the findings of this study are openly available in the Open Science Framework (OSF) at: https://osf.io/qb6sd/overview.
