# Supplementary material for: Electrocortical and Physiological Correlates of Memory and Attention During the Anticipation of Avoidable and Inevitable Threats
Source: Psychophysiology. 2026 Sep 27;63(10):e70413. doi: 10.1111/psyp.70413 (PMC13617175; doi:10.1111/psyp.70413)
Supplement: Supplementary file 1 [file PSYP-63-e70413-s001.docx]

**Supplemental Materials for**

**Electrocortical and Physiological Correlates of Memory and Attention during the Anticipation of Avoidable and Inevitable Threats**

Yannik Stegmann^1^, Matthias Gamer^1^

^1^ Department of Psychology, University of Würzburg, 97070 Würzburg, Germany

**Author Note**

Yannik Stegmann <https://orcid.org/0000-0002-0933-8492>

Matthias Gamer <https://orcid.org/0000-0002-9676-9038>

Correspondence concerning this article should be addressed to Yannik Stegmann, Department of Psychology, University of Würzburg, Marcusstraße 9-11, 97070 Würzburg, Germany. Email: [Yannik.stegmann@uni-wuerzburg.de](mailto:Yannik.stegmann@uni-wuerzburg.de), Phone: +49 931 31-84357

**Analysis of the temporal dynamics of the response pattern**

To analyze the temporal dynamics of the response pattern, trials were divided into three equal bins corresponding to thirds of the experiment. Each bin contained up to 30 trials per condition, which were further categorized based on whether faces were subsequently remembered or forgotten. Consequently, the number of analyzable trials varied substantially across participants (range: 1 - 29). We then repeated all analyses including an additional factor, *phase* (three levels: first, second, and third third of the experiment). Please note, that we were unable to conduct the corresponding analyses for changes in alpha activity, as the combination of classification factors (i.e., cue, memory, and phase) resulted in a substantial number of empty cells across participants. Consequently, only 27 (< 50 %) participants had sufficient data in all conditions, which was deemed inadequate for comparable statistical analysis.

**Heart Rate**

The Memory × Cue × Phase ANOVA for the time window of the final second before the facial stimulus offset revealed a significant main effect of Cue, *F*(1.49, 87.67) = 78.45, *p* < .001, $\eta_{p}^{2}$ = .57, *BF_10_* = 2.39 e+69 and a significant interaction between cue, memory, and phase, *F*(4, 236) = 3.70, *p* = .006, $\eta_{p}^{2}$ = .06, *BF_int_* = 3.66 e-08. A visual inspection of Figure S1 indicates mostly stable heart rate response patterns across the 270 trials of the experiment, while heart rate deceleration during inevitable threat trials that are subsequently forgotten seemed reduced at the end of the experiment. All other effects were not significant, *ps* > .301, *BFs* < .007.

Figure S1. Heart rate changes (bpm) over time for inevitable (red), avoidable threat (yellow), and safety (green) trials, separated by subsequently remembered (solid lines) versus forgotten (dashed lines) faces and bins corresponding to thirds of the experiment (first, second, and third third of the experiment). Error bars represent ±1 SEM with shaded areas indicating pre-registered analysis windows. Vertical dashed lines represent the onset and offset of the neutral facial expressions, respectively.

**Pupillary Responses**

*Cue Onset.* The Memory × Cue × Phase ANOVA for the cue onset revealed significant main effects of cue, *F*(2, 118) = 16.77, *p* < .001, $\eta_{p}^{2}$ = .22, *BF_10_* = 1.60 e+18, and phase, *F*(2, 118) = 15.88, *p* < .001, $\eta_{p}^{2}$ = .21, *BF_10_* = 1.90 e+6, which are further qualified by a Cue × Phase interaction, *F*(4, 236) = 8.24, *p* < .001, $\eta_{p}^{2}$ = .10, *BF_int_* = 144.79, and a marginally significant Memory × Phase interaction, *F*(2, 118) = 2.77, *p* = .067, $\eta_{p}^{2}$ = .04, *BF_int_* =.46. All other effects are not significant, ps > .105, *BFs* < .22.

*Face Onset.* For the onsets of the faces, the ANOVA showed significant effects of cue, *F*(2, 118) = 19.91, *p* < .001, $\eta_{p}^{2}$ = .25, *BF_10_* = 7.22 e+13, phase, *F*(2, 118) = 17.99, *p* < .001, $\eta_{p}^{2}$ = .23, *BF_10_* = 2.01 e+7, and their interaction, *F*(3.42, 201.91) = 4.74, *p* =.002, $\eta_{p}^{2}$ = .07, *BF_int_* = 4.04. In addition, there was a significant interaction between phase and memory, *F*(2, 118) = 3.31, *p* = .040, $\eta_{p}^{2}$ = .05, *BF_int_* = .24, and a marginal interaction between cue and memory, *F*(2, 118) = 2.60, *p* = .078, $\eta_{p}^{2}$ = .04, *BF_int_* = .14. All other effects are not significant, ps > .132, *BFs* < .17

*Trial End.* For pupil diameter changes at the end of the trials, Next to the main effects of cue, *F*(1.44, 85.24) = 65.50, *p* < .001, $\eta_{p}^{2}$ = .53, *BF_10_* = 3.32 e+64, phase, *F*(2, 118) = 4.32, *p* = .015, $\eta_{p}^{2}$ = .07, *BF_10_* = .48, and memory, *F*(1, 59) = 21.32, *p* < .001, $\eta_{p}^{2}$ = .27, *BF_10_* = 193.08, there were significant interactions between cue and phase, *F*(3.44, 202.72) = 7.47, *p* < .001, $\eta_{p}^{2}$ = .11, *BF_int_* = 93.44, and between cue, memory, and phase, *F*(4, 236) = 3.11, *p* = .016, $\eta_{p}^{2}$ = .05, *BF_int_* = .25.

The temporal dynamics are depicted in Figure S2. The significant interactions between cue and phase indicate that differences between conditions decrease over the course of the experiment, particularly for responses at cue and face onset. This pattern is consistent with habituation processes, potentially reflecting reduced arousal over time. Importantly, the overall pattern of results remains largely stable, especially for the avoidable threat condition. Visual inspection of Figure S2 further suggests that the significant three-way interaction at the end of the trial is driven by stronger memory-related effects for avoidable threat and safety trials early in the experiment, which diminish over time. In contrast, the memory effect for avoidable threat trials appears relatively robust across phases.

Figure S2. Changes in pupil diameter over time for inevitable (red), avoidable threat (yellow), and safety (green) trials, separated by subsequently remembered (solid lines) versus forgotten (dashed lines) faces and bins corresponding to thirds of the experiment (first, second, and third third of the experiment). Error bars represent ±1 SEM with shaded areas indicating pre-registered analysis windows. Vertical dashed lines represent the onset and offset of the neutral facial expressions, respectively.

**Oculomotor Responses**

*Center bias.* The ANOVA for the distance to the center of the screen for the last second before trial offset revealed a significant main effect of cue, *F*(1.61, 85.43) = 11.63, *p* < .001, $\eta_{p}^{2}$ = .18, *BF_10_* = 4.64 e+3, that is further qualified by a Cue × Phase interaction, *F*(4, 212) = 6.04, *p* < .001, $\eta_{p}^{2}$ = .10, *BF_int_* = 503.31. Other effects were not significant, *ps* > .159, *BFs* < .09

D*uration of fixations.* Similarly, the ANOVA for the duration of fixations during the last second showed a significant main effect of cue, *F*(1.12, 59.75) = 56.75, *p* < .001, $\eta_{p}^{2}$ = .52, *BF_10_* = 2.23 e+42, that is further qualified by a Cue × Phase interaction, *F*(2.46, 130.64) = 27.97, *p* < .001, $\eta_{p}^{2}$ = .35, *BF_int_* = 2.55 e+21. Other effects were not significant, *ps* > .125, *BFs* < .07.

*Number of fixations.* Likewise, the ANOVA for the duration of fixations during the last second showed a significant main effect of cue, *F*(1.45, 77.05) = 42.96, *p* < .001, $\eta_{p}^{2}$ = .45, *BF_10_* = 1.85 e+23, that is further qualified by a Cue × Phase interaction, *F*(2.99, 158.32) = 14.28, *p* < .001, $\eta_{p}^{2}$ = .21, *BF_int_* = 4.68 e+06. Other effects were not significant, *ps* > .173, *BFs* < .07.

In summary, the results for indices of oculomotor responses suggest that differences between conditions were robust during the first 180 trials but largely diminished in the final third of the experiment (see Figure S3). This pattern is consistent with reduced arousal or increasing fatigue over time and aligns with our initial predictions regarding eye movements toward faces in this paradigm.

Figure S3. Oculomotor responses as a function of memory outcome, cue condition, and third of experiment. (A) Distance from the center [in pixels] over time for inevitable (red), avoidable threat (yellow), and safety (green) trials, separated by subsequently remembered (solid lines) versus forgotten (dashed lines) faces. (B) Duration and (C) number of fixations over time for the same conditions. Error bars represent ±1 SEM with shaded areas indicating the analysis windows. Vertical dashed lines represent the onset and offset of the neutral facial expressions, respectively.

**Supplementary Analysis Using a More Liberal Memory Criterion**

To examine memory effects beyond high-confidence recognition, we applied a more liberal cutoff criterion (> 0) to classify trials as remembered versus forgotten. As expected, this approach resulted in fewer forgotten trials (see Table S1), and a total of nine participants had to be excluded due to missing cells in at least one combination of Cue × Memory. Because the main effects of condition remained unchanged relative to the analyses reported in the main manuscript, we focus here on reporting the effects of memory.

Table S1. Trials (*M ± SD*) available for analyses using the more liberal memory criterion

| Condition | Inevitable Threat | Avoidable Threat | Safety |
| --- | --- | --- | --- |
| Remembered | 45.9 ± 12.7 | 47.4 ± 14.0 | 44.2 ± 11.2 |
| Forgotten | 16.6 ± 10.9 | 16.4 ± 12.7 | 15.9 ± 10.5 |

***Memory ratings***

Using the more liberal memory criterion, participants classified a greater number of faces as previously seen (see Figure S4). However, there were still no significant differences between the three threat conditions in the number of correctly remembered faces, *F*(1.63, 87.81) = .86, *p* = .405, $\eta_{p}^{2}$ = .02, *CI_95_* = [.00; .08], *BF_10_* = .13.

Figure S4. Memory performance across experimental conditions expressed as percentage of correctly recognized faces for each condition. Bars depict *means ± SEM* with individual data points shown. Participants demonstrated better memory for previously seen faces relative to novel faces.

**Alpha Activity**

*Cue Onset.* As for the main analysis, the Memory × Cue ANOVA for the fixation-cross period revealed a significant main effect of Memory, *F*(1, 52) = 10.29, *p* = .002, $\eta_{p}^{2}$ = .17, *CI_95_* = [.02; .34], *BF_10_* = 8.02, indicating stronger alpha suppression for subsequently remembered faces already during the presentation of the colored fixation cross signaling the trial type. In addition, there was a significant main effect of Cue, *F*(2, 104) = 4.94, *p* = .009, $\eta_{p}^{2}$ = .09, *CI_95_* = [.01; .19], *BF_10_* = 8.07. The Memory × Cue interaction was not significant, *F*(2, 104) = .15, *p* = .863, $\eta_{p}^{2}$ < .01, *CI_95_* = [.00; .03], *BF_int_* = .07.

*Face Onset.* In the time window following the face onset, the ANOVA again revealed a significant main effect of Memory, *F*(1, 52) = 8.80, *p* = .005, $\eta_{p}^{2}$ = .14, *CI_95_* = [.02; .32], *BF_10_* = 6.90, with stronger alpha suppression for subsequently remembered faces. The main effect of Cue was also significant, *F*(2, 104) = 6.28, *p* = .003, $\eta_{p}^{2}$ = .11, *CI_95_* = [.02; .22], *BF_10_* = 23.03, but not the Memory × Cue interaction, *F*(2, 104) = 1.00, *p* = .371, $\eta_{p}^{2}$ = .02, *CI_95_* = [.00; .08], *BF_int_* = .12.

*Trial End.* During the final second before the offset of the neutral face, the ANOVA again showed significant main effects of Memory, *F*(1, 52) = 11.81, *p* = .001, $\eta_{p}^{2}$ = .19, *CI_95_* = [.03; .36], *BF_10_* = 10.50, and Cue, *F*(2, 104) = 12.91, *p* < .001, $\eta_{p}^{2}$ = .20, *CI_95_* = [.07; .32], *BF_10_* = 17468.99. The Memory × Cue interaction was again non-significant, *F*(2, 104) = .15, *p* = .860, $\eta_{p}^{2}$ < .01, *CI_95_* = [.00; .03], *BF_int_* = .07, suggesting generally stronger alpha suppression for subsequently remembered versus forgotten faces independent of the condition.

In summary, the results were fully consistent with the main findings when applying the more liberal memory criterion. All EEG results are presented in Figure S5.

Figure S5. Changes in alpha activity as a function of memory outcome and cue condition over time for inevitable threat (red), avoidable threat (yellow), and safety (green) trials, separated by subsequently remembered (solid lines) versus forgotten (dashed lines) faces. Error bars represent ±1 SEM with shaded areas indicating pre-registered analysis windows. Vertical dashed lines represent the onset and offset of the neutral facial expressions, respectively.

**Heart Rate**

As for the main analyses, the Memory × Cue ANOVA for the final second before the facial stimulus offset revealed only a significant main effect of Cue, *F*(1.45, 78.37) = 60.88, *p* < .001, $\eta_{p}^{2}$ = .53, *CI_95_* = [.40; .62], *BF_10_* = 5.44 e+30. Effects including memory were not significant, *ps* > .300, *BFs* < .14. Changes in heart rate are depicted in Figure S6A.

**Pupillary Responses**

*Cue Onset.* Like in the main analysis, the Memory × Cue ANOVA for the cue onset revealed a significant main effect of condition, *F*(2, 108) = 21.56, *p* < .001, $\eta_{p}^{2}$ = .28, *CI_95_* = [.14; .40], *BF_10_* = 3.17 e+10, which is further qualified by a Memory × Cue interaction, *F*(2, 108) = 3.72, *p* = .027, $\eta_{p}^{2}$ = .06, *CI_95_* = [.00; .16], *BF_int_* = .33. Follow-up t-tests showed stronger pupil dilation for later forgotten compared to remembered faces only for inevitable threat trials, *t*(54) = 3.14, *p* = .003, *d* = .42, *CI_95_* = [.15; .70], *BF_10_* = 11.30, but not for avoidable threat, *t*(54) = .19, *p* = .848, *d* = .03, *CI_95_* = [-.24; .29], *BF_10_* = .14, or safety trials, *t*(54) = .27, *p* = .790, *d* = .04, *CI_95_* = [-.23; .30], *BF_10_* = .15.

*Face Onset.* In the window following face onset, the ANOVA revealed a significant main effect of Cue, *F*(2, 108) = 19.90, *p* < .001, $\eta_{p}^{2}$ = .27, *CI_95_* = [.13; .39], *BF_10_* = 2.62 e+9, and Memory, *F*(1, 54) = 11.49, *p* < .001, $\eta_{p}^{2}$ = .18, *CI_95_* = [.03; .35], *BF_10_* = 2.04, and a marginal Memory × Cue interaction, *F*(2, 108) = 2.76, *p* = .068, $\eta_{p}^{2}$ = .05, *CI_95_* = [.00; .14], *BF_int_* = .35. Here, the current results deviate from the main analysis by already showing generally stronger pupil dilation for later forgotten compared to remembered faces; however, this finding is consistent with the original results at the end of the trials.

*Trial End.* Again, as for the main analyses, the ANOVA for pupil diameter changes at the end of the trials, revealed a significant main effect of Cue, *F*(1.49, 79.84) = 50.96, *p* < .001, $\eta_{p}^{2}$ = .49, *CI_95_* = [.34; .59], *BF_10_* = 7.67 e+27, and Memory, *F*(1, 54) = 29.70, *p* < .001, $\eta_{p}^{2}$ = .35, *CI_95_* = [.16; .51], *BF_10_* = 50.33, but no Memory × Cue interaction, *F*(1.75, 94.57) = 2.28, *p* = .114, $\eta_{p}^{2}$ = .04, *CI_95_* = [.00; .12], *BF_int_* = .16.

In summary, using a more liberal criterion for memory scores did not substantially change the pattern of results for changes in pupil diameter. All results are depicted in Figure S6B.

Figure S6. Autonomic responses as a function of memory outcome and cue condition. (A) Heart rate change (bpm) over time for inevitable threat (red), avoidable threat (yellow), and safety (green) trials, separated by subsequently remembered (solid lines) versus forgotten (dashed lines) faces. (B) Pupil diameter change (mm) over time for the same conditions. Error bars represent ±1 SEM with shaded areas indicating pre-registered analysis windows. Vertical dashed lines represent the onset and offset of the neutral facial expressions, respectively.

**Oculomotor Responses**

*Center bias.* The Memory × Cue ANOVA for gaze distance to the center of the screen during the final second of the trial revealed a main effect of Cue, *F*(1.56, 77.86) = 4.10, *p* = .029, $\eta_{p}^{2}$ = .08, *CI_95_* = [.00; .18], *BF_10_* = 2.89, and a main effect of Memory, *F*(1, 50) = 5.00, *p* = .030, $\eta_{p}^{2}$ = .09, *CI_95_* = [.00; .26], *BF_10_* = .72, but no Memory × Cue interaction, *F*(2, 100) = .83, *p* = .441, $\eta_{p}^{2}$ = .02, *CI_95_* = [.00; .08], *BF_10_* = .13 (see Figure S7A). The exploratory analysis of the full trial excluding the last second confirmed the main effect of Memory, *F*(1, 50) = 17.33, *p* < .001, $\eta_{p}^{2}$ = .26, *CI_95_* = [.07; .43], *BF_10_* = 15.85, while the interaction became marginally significant, *F*(2, 100) = 3.05, *p* = .052, $\eta_{p}^{2}$ = .06, *CI_95_* = [.00; .15], *BF_10_* = .37, indicating a weaker center bias for subsequently remembered versus forgotten faces, especially for the inevitable and safety trials (see Figure S7A).

*Duration of fixations.* The ANOVA for the final second of the trial revealed again only a main effect of Cue, *F*(1.14, 56.97) = 46.37, *p* < .001, $\eta_{p}^{2}$ = .48, *CI_95_* = [.33; .58], *BF_10_* = 1.81 e+25, but no main effect of Memory, *F*(1, 50) = .2.03, *p* = .160, $\eta_{p}^{2}$ = .04, *CI_95_* = [.00; .18], *BF_10_* = .17, and no Memory × Cue interaction, *F*(1.57, 78.65) = 1.87, *p* = .169, $\eta_{p}^{2}$ = .04, *CI_95_* = [.00; .12], *BF_int_* = .12 (see Figure S7B). As in the main analysis, the exploratory full-trial analysis (excluding the final second) revealed a main effect of Memory, *F*(1,50) = 5.67, *p* = .021, $\eta_{p}^{2}$ = .10, *CI_95_* = [.00; .27], *BF_10_* = .37, with shorter fixations for subsequently remembered versus forgotten faces. The interaction was again non-significant, *F*(1.71, 85.71) = .03, *p* = .958, $\eta_{p}^{2}$ < .01, *CI_95_* = [.00; .00], *BF_int_* = .06.

*Number of fixations.* Likewise, the ANOVA for the number of fixations during the final second revealed a main effect of Cue, *F*(1.56, 77.87) = 31.31, *p* < .001, $\eta_{p}^{2}$ = .39, *CI_95_* = [.23; .50], *BF_10_* = 6.28 e+13, but no main effect of Memory, *F*(1, 50) = .09, *p* = .761, $\eta_{p}^{2}$ < .01, *CI_95_* = [.00; .08], *BF_10_* = .13, and no Memory × Cue interaction, *F*(2, 100) = 1.17, *p* = .314, $\eta_{p}^{2}$ = .02, *CI_95_* = [.00; .19], *BF_int_* = .13 (see Figure S7C). Exploratory full-trial analysis (excluding the final second) again revealed a main effect of Memory, *F*(1,50) = 8.08, *p* = .007, $\eta_{p}^{2}$ = .11, *CI_95_* = [.01; .22], *BF_10_* = 2.22, indicating more fixations for subsequently remembered versus forgotten faces, while the interaction was non-significant, *F*(2,100) = 1.75, *p* = .179, $\eta_{p}^{2}$ = .03, *CI_95_* = [.00; .11], *BF_int_* = .15.

In summary, the results for oculomotor indices using the more liberal memory criterion replicated the findings of the main analysis. Differences were observed only for the center bias index, indicating that a weaker center bias was associated with better memory encoding, including during the final second of the trial.

Figure S7. Oculomotor responses as a function of memory outcome and cue condition. (A) Distance from the center [in pixels] over time for inevitable threat (red), avoidable threat (yellow), and safety (green) trials, separated by subsequently remembered (solid lines) versus forgotten (dashed lines) faces. (B) Duration and (C) number of fixations over time for the same conditions. Error bars represent ±1 SEM with shaded areas indicating the analysis windows. Vertical dashed lines represent the onset and offset of the neutral facial expressions, respectively.
